# Strategies to Improve Meat Quality and Safety

**DOI:** 10.1155/2016/9523621

**Published:** 2016-07-05

**Authors:** Agostino Sevi, Rosaria Marino, José M. Lorenzo, Brigitte Picard, Angelica Simone Cravo Pereira

**Affiliations:** ^1^Department of Agricultural Food and Environmental Sciences, University of Foggia, 71122 Foggia, Italy; ^2^Centro Tecnológico de la Carne de Galicia, Rúa Galicia No. 4, Parque Tecnológico de Galicia, San Cibrán das Viñas, 32900 Ourense, Spain; ^3^INRA-Clermont-Ferrand-Theix-Lyon Centre, 63122 Saint-Genes-Champanelle, France; ^4^Faculdade de Medicina Veterinária e Zootecnia, University of Sao Paulo, 05508-270 Sao Paulo, SP, Brazil

Meat quality refers to intrinsic attributes critical for the suitability of meat for eating, processing, and storage, including retail display. The main attributes of interest of meat are safety, nutritional value, sensorial properties, lipid composition, oxidative stability, and consistency.

In recent years the meat market has undergone changes that require high standards of quality, so aspects related to environmental sustainability and animal welfare have become critical in meat production. In addition, there is growing awareness of the link between diet and health and this is reflected in the demand for more information and for products which are healthy and of consistently high quality. Therefore, meat quality and safety are becoming dynamic and challenging concerns which require the generation of new information and of continuous reevaluation of existing knowledge for meeting market's demands by assuring high quality standards and prevention of recognized risks to human health.

The papers included in this issue cover aspects of muscle biology and meat biochemistry and discuss factors affecting meat quality and sensory properties, contribution of beef to human health.

For meat research, a main objective is to control concomitantly the development of muscles and the qualities of meat commercial cuts from different animal species. In the paper “Expression Marker-Based Strategy to Improve Beef Quality” the steps of an expression marker-based strategy to improve beef sensory quality were described, from the discovery of biomarkers that identify consistent beef and the biological functions governing beef tenderness to the integration of the knowledge into detection tests for desirable animals. The review “How Muscle Structure and Composition Influence Meat and Flesh Quality” describes the features of muscle components and their relationships with the technological, nutritional, and sensory properties of meat/flesh from different livestock and fish species.

The effect of diet and rearing system on meat quality was studied in two different studies. Particularly, the research “Influence of Maternal and Postweaning Linseed Dietary Supplementation on Growth Rate, Lipid Profile, and Meat Quality Traits of Light Sarda Lambs” highlights that linseed supplementation and early life nutrition can influence performance and fatty acids metabolism in growing lambs. In the study “A Survey on the Effect of Livestock Production System and Finishing Diet on Sensory Characteristics of Foal Meat Using Generalized Procrustes Analysis” highly appreciated sensory properties were mostly associated with foals from the semiextensive system.

Finally, the important role of meat and meat products as source of protein, fat, and several functional compounds was highlighted in the review “A Contribution of Beef to Human Health: A Review of the Role of the Animal Production Systems.”

We hope that all the interesting results published in this special issue will stimulate further studies to improve meat quality and safety.

*Agostino Sevi* *Agostino Sevi* *Rosaria Marino* *Rosaria Marino* *José M. Lorenzo* *José M. Lorenzo* *Brigitte Picard* *Brigitte Picard* *Angelica Simone Cravo Pereira* *Angelica Simone Cravo Pereira*

